# Usage of fractional order ${\textrm{PI}}^\lambda {\textrm{D}}^\mu$ controller as AQM algorithm

**DOI:** 10.1038/s41598-023-45667-2

**Published:** 2023-10-28

**Authors:** Karol Marszałek, Adam Domański, Adam Milik

**Affiliations:** 1 Department of Distributed Systems and Informatic Devices, Silesian University of Technology, Gliwice, 44-100 Poland; 2 Division of Digital Systems, Silesian University of Technology, Gliwice, 44-100 Poland

**Keywords:** Computer science, Information technology, Software, Electrical and electronic engineering

## Abstract

The article describes the Usage of fractional order ${\textrm{PI}}^\lambda {\textrm{D}}^\mu$ controller as AQM algorithm. The alternative, integer-based calculation process for ${\textrm{PI}}^\lambda {\textrm{D}}^\mu$ controller is proposed and tested in numerical analysis, simulation environment, and Linux-based testbed environment with real-life devices. The FPGA design for the calculation process is presented. Experimental evaluation and setup process for AQM in the network is presented.

## Introduction

With the development of IT systems comes an increased need for efficient communication capabilities. With more and more devices interconnected via computer networks and the rising popularity of streaming services, new challenges to ensuring the quality of services arose. Among others, one of the challenges is a congestion problem. Computer network traffic is transferred in the form of packets via links with limited bandwidth. If the number of packets received by a router during some period is bigger than the transmission capabilities on the other interface, which is typically the case, the packets need to be stored in the devices in order to wait for the transmission. The buffer in the devices in which packets are stored is called a queue. Growing queue sizes cause problems called *bufferbloat*^1^. Essentially, bufferbloat causes increasing delays in the network.

To deal with the problem of increasing delays in the network caused by the bufferbloat problem, the* Internet Engineering Task Force (IETF) * recommends usage of routers implementing *Active Queue Management (AQM)*. The idea of AQM mechanisms is to provide a way of employment of congestion control algorithms implemented in TCP transmitters. TCP protocol uses a congestion window mechanism to adjust the number of packets sent via a link over time based on the number of packets that have been dropped during the transmission. When the packet is dropped, the TCP transmitter slows down and gradually speeds up until the next packet is dropped. However, this leads to the global synchronization problem when the transmitters change the transmission window at a similar moment due to dropping packets. The AQM mechanism is based on the idea of randomly dropping packets with growing congestion in the network. By dropping packets earlier, the transmitters are notified about congestion at random moments, solving the global synchronization problem and decreasing delays in the network^2^.

Among all of the currently existing AQM mechanisms, the most known is the *Random Early Detection (RED)* proposed by S. Floyd and V. Jacobson^3^. The idea was based on the moving queue length average. At the moment of packet arrival, the moving average of queue length is used to calculate the dropping probability of the packet—the longer the queue, the bigger the probability of the packet being dropped. The mechanism was proven to help control the delay in the network.

Since RED AQM was presented, many other solutions have been created. The new solutions were either the improvements of the RED algorithm^4^ or the new ideas based on the different probability calculation mechanisms. There is a variety of solutions using artificial intelligence^5^^6^^7^^8^. The attempt to combine the Explicit Congestion Notification (ECN) with machine learning techniques is presented in^9^. There are already known AQM mechanisms that utilize the PI controller^10^, and other works present the attempts of usage of PID controller^11^. Several AQM solutions were proposed to deal with the congestion problem under more specific circumstances, like in 5G networks^12^, other wireless networks^13^, or in real-time video streaming^14^.

The evaluation of the AQM algorithms is typically done with one or more approaches: using numerical analysis^15^, simulation^16^, or experimental evaluation in physical testbed^17^. To model network dynamics, commonly the Fluid-Flow^18^ and diffusion approximation frameworks are used^19^. To create the simulation, the SimPy^20^, NS-2^21^ and NS-3^22^ frameworks can be used. In this paper, we present the experimental evaluation of the AQM mechanism based on the fractional order ${\textrm{PI}}^\lambda {\textrm{D}}^\mu$ controller. We use the SimPy-based simulation and physical testbed.

The use of fractional order ${\textrm{PI}}^\lambda {\textrm{D}}^\mu$ controller as active queue management algorithm requires adaptation of calculation process to capabilities and computing power of small devices typically used as network routers. Since growing calculation time negatively impacts the solution’s overall efficiency, it must be maintained at the lowest possible level. Fractional order ${\textrm{PI}}^\lambda {\textrm{D}}^\mu$ controller requires a significant amount of floating point calculations to be performed to calculate the controller’s value at the given moment. A considerable number of floating point operations tend to be time-consuming, especially for devices lacking hardware support for them. To overcome this problem, we have created a solution that calculates the response value of ${\textrm{PI}}^\lambda {\textrm{D}}^\mu$ controller using only integers. Eliminating the floating point operations makes the calculation process faster but with limited precision.

Besides using integer-based calculation instead of floating point operations, other methods are available to shorten the calculation time. The paper^23^ presents the implementation of ${\textrm{PI}}^\lambda {\textrm{D}}^\mu$ controller using GPU to boost the calculation time. In this paper, we present the implementation of ${\textrm{PI}}^\lambda {\textrm{D}}^\mu$ controller using dedicated *Field Programmable Gate Arrays (FPGA)* design to calculate the response values of the controller. The FPGA-based designs to calculate the response of PID controllers are present in various fields like motion control systems^24^, respiratory systems^25^ or industrial PLC controllers^26^. Among currently existing solutions, there are also already taken attempts to implement a fractional-order ${\textrm{PI}}^\lambda {\textrm{D}}^\mu$ controller with FPGA^27^. Since tuning the controller is a key to its efficiency, there are attempts to create a controller with parameter adjustment capabilities^28^. The usage of FPGA has the potential to improve the calculation time and accuracy. There is still a trade-off between calculation speed and accuracy. The FPGA design can either speed up the calculations or keep the speed acceptable (compared to other devices, like routers we used in the experiments) while improving the calculation accuracy.

The goal of the paper was to implement the AQM algorithm in the real-life networking device based on the ${\textrm{PI}}^\lambda {\textrm{D}}^\mu$ controller. Most of the devices used as networking equipment have limited calculation capabilities. The goal of the research presented in the paper is recognition of implementation limitations related to the usage of fractional order ${\textrm{PI}}^\lambda {\textrm{D}}^\mu$ controller as AQM mechanism. As part of research, we implemented the AQM algorithm based on the idea of fractional order ${\textrm{PI}}^\lambda {\textrm{D}}^\mu$ controller in OpenWRT system. The created implementation was compiled for device Mikrotik Routerboard RB951UI-2HND and tested in both simulation and Linux-based testbed environments. We have also created a dedicated FPGA design to speed up the calculation process used in FPGA-based routers.

The rest of the article looks as follows: Section “Theoretical background” presents the theoretical background for the research. Subsection “Simulation model of network node” presents the model used in simulation and subsection [Sec Sec4] presents the fractional order PID controller as AQM mechanism. Section “Dedicated FPGA design” describes the FPGA design we propose for calculation of the controller’s response in FPGA-based router. Section “Methods” presents the experiment’s methodology, and Section “Results” presents the results of all conducted experiments. The last section, Section “Conclusions”, gathers the conclusions.

## Theoretical background

This section presents the theoretical background for our research. First, we discuss the model which we use in the simulation. The model is a close approximation of a single real-life router we use in our testbed environment. Both, the model and device we use are based on the single packets queue. The second part of the section presents the method of calculation the response of ${\textrm{PI}}^\lambda {\textrm{D}}^\mu$ controller used as AQM mechanism.

### Simulation model of network node

**Figure 1:**
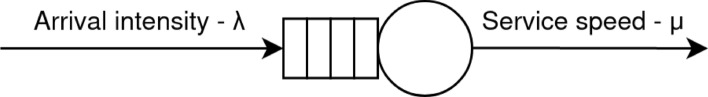
Network node model used in simulation.

The description of the AQM queue was presented in^29^ as an example of an IoT gateway. In general, we model the system incorporating the AQM mechanism as the queue with the controller decides about the packet acceptance or rejection. Graphical representation of the model we use is presented in Fig. 1.

In the presented model, there are two key parameters. The $\lambda$ is the rate of packets arrivals—the average number of packets incoming to the system per unit of time. The $\mu$ is the rate of packets service—the average number of packets leaving the device per unit of time. When the rate of receiving packets ($\lambda$) is bigger than the rate of sending packets out of the queue ($\mu$), the queue in the device grows. On each event of the enqueuing packet, the AQM algorithm calculates the probability of dropping it based on the queue length. Based on this probability, the packet is accepted or rejected. In standard solutions, the dropping probability function is a typical mathematical function, for example, linear or exponential. We replace the probability function with the response of ${\textrm{PI}}^\lambda {\textrm{D}}^\mu$ controller. The controlled signal is the queue length.

### ${\textrm{PI}}^\lambda {\textrm{D}}^\mu$ controller as AQM mechanism

The purpose of ${\textrm{PI}}^\lambda {\textrm{D}}^\mu$ controller is to keep the value of the controlled signal on the given level. The non-integer order controllers have been proven better at this job in earlier works^30^. In order to keep the signal at the desired level, the error value from the current value of the signal and the desired one is calculated as a difference between the two. In order to use ${\textrm{PI}}^\lambda {\textrm{D}}^\mu$ controller as AQM mechanism as a controlled signal we use the current length of the packets queue in the router. In the standard PID controller, the integral part is a simple sum of historical queue lengths (Eq. 5). Similarly, the derivative part is the difference between the current and previous queue lengths (Eq. 4). Both the integral and derivative parts are calculated in the same way. The calculated value is derivative for positive order, while for the negative order, it is an integral. Equations (6) and (7) show that in the case of fractional order calculus, instead of a simple sum, we get the sum of the queue length multiplied by the coefficients related to integral and derivative order. Those coefficients improve the performance of the AQM mechanism by improving its adjustment to traffic conditions.

A description of how to use the response of the ${\textrm{PI}}^\lambda$ controller is presented in^31^. The probability of dropping the packets on arrival is given by the controller response and is given by the Eq. (1).

1 $$\begin{aligned} p = max(0, -( K_{p}e_{0} + K_{i}\sum _{j\in V_{i} }\nu _{i}(j)e_{j} +K_{d}\sum _{j\in V_{d} }\nu _{d}(j)e_{j} ) ) \end{aligned}$$

where:K_p_—amplification of proportional part, K_i_—amplification of an integral part, K_d_—amplification of derivative part,e$_{\textrm{j}}$ = q$_{\textrm{j}}$—setpoint—the value of j-th error, setpoint—the desired queue length,q$_{\textrm{j}}$—j-th queue length,V_i_—set of integral part coefficients,V_d_—set of derivative part coefficients.

Elements of the sets V_i_ and V_d_ are given as:

2 $$\begin{aligned} \nu _{i}(j) =\ {\left\{ \begin{array}{ll} 1&{}j=0 \\ \nu _{i}(j-1)(1 - (\frac{1+\gamma }{j})) &{} j>0 \end{array}\right. } \end{aligned}$$

where $\gamma$ = $\lambda$ for an integration order or $\gamma$ = $\mu$ for a derivation order.

The packet management in the AQM routers is done at the discrete points at the time of packet arrival. Therefore, we consider the queue model as a case of discrete systems. We use Grunwald-Letnikov’s definition of the discrete differ-integrals of fractional order in our calculations.

For a given sequence f$_{0}$, f$_{1}$,…, f$_{\textrm{j}}$,…, f$_{\textrm{k}}$:

3 $$\begin{aligned} \Delta ^{\gamma } f_{k} = \sum ^{k}_{j=0} (-1)^{j} \genfrac(){0.0pt}0{\gamma }{j} f_{k-j} \end{aligned}$$

where $\gamma \in {\textbf{R}}$ is a non-integer, fractional order, f$_{\textrm{k}}$ is a differentiated discrete function and $\genfrac(){0.0pt}0{\gamma }{j}$ is generalized Newton symbol.

For $\gamma = 1$, we get the formula for the first-order derivative:

4 $$\begin{aligned} \Delta ^{1} x_{k} = 1x_{k} - 1x_{k-1} + 0x_{k-2} + 0x_{k-3}... \end{aligned}$$

For $\gamma = -1$, we get the formula for the first-order discrete integral:

5 $$\begin{aligned} \Delta ^{-1} x_{k} = 1x_{k} + 1x_{k-1} + 1x_{k-2} + 1x_{k-3}... \end{aligned}$$

For a fractional-order derivative and integral order, we get the weighted sum of all samples, for example:

6 $$\begin{aligned} \Delta ^{-1.2} x_{k} = 1x_{k} + 1.2x_{k-1} + 1.32x_{k-2} + 1.408x_{k-3}... \end{aligned}$$

7 $$\begin{aligned} \Delta ^{-0.8} x_{k} = 1x_{k} + 0.8x_{k-1} + 0.72x_{k-2} + 0.672x_{k-3}... \end{aligned}$$

The calculations presented above require a significant number of floating point operations, which are problematic to perform on devices with limited calculational capabilities. To overcome the problem of floating point operations, the approach proposed further in the document estimates the ${\textrm{PI}}^\lambda {\textrm{D}}^\mu$ controller value using only integer numbers in the calculation process. The main idea behind the alternative calculation process may be expressed as follows: using the bit shift, an integer number can approximate a fractional number with the given resolution. In the proposed solution, all controller parameters are integer numbers with an assumed shift of 13 bits to the left. The shift value of 13 bits allows to set the controller parameters with the resolution of 1/2$^{13}$. For instance, usage of value 1 means that we are using a parameter of value 1/2$^{13}$, and usage of value 2$^{13}$ is equal to 1.0. Because in the calculation process, at most two parameters are multiplied, calculated ${\textrm{PI}}^\lambda {\textrm{D}}^\mu$ controller value is equal to the actual value, which would be obtained using floating point operations, and the value of 2$^{26}$. The drawback of the proposed approach is the calculation error, especially for values close to the resolution value or smaller. Due to this fact, it is needed to determine the calculation error impact on the overall controller efficiency. The calculation error also influences the length of the error history. The longer the error history, the smaller values are used in the calculation process, and the error grows, which cancels out the benefits of remembering more historical errors.

## Dedicated FPGA design

Besides the router’s implementation, we present the solution based on Field Programmable Gate Arrays (FPGA). In our work, we present the FPGA design for performing the ${\textrm{PI}}^\lambda {\textrm{D}}^\mu$ controller calculations that can be incorporated in the FPGA router design. The proposed solution is based on a NetFPGA FPGA-1 G-CML development platform with a Xilinx Kintex-7 FPGA. The hardware project was prepared using the Xilinx Vivado^®^ Design Suite development environment. The FPGA includes four 1 Gb/s Ethernet interfaces with auto-negotiation and a PCI-Express expansion bus. Therefore, the board is capable of working as a network router. When a network packet is received on one of the interfaces, the presented module calculates the dropping probability for this packet. Based on the calculated value, the packet is either accepted, enqueued in the device, and eventually sent via destination interfaces or dropped with negative acknowledgment sent back to the sender.

**Figure 2:**
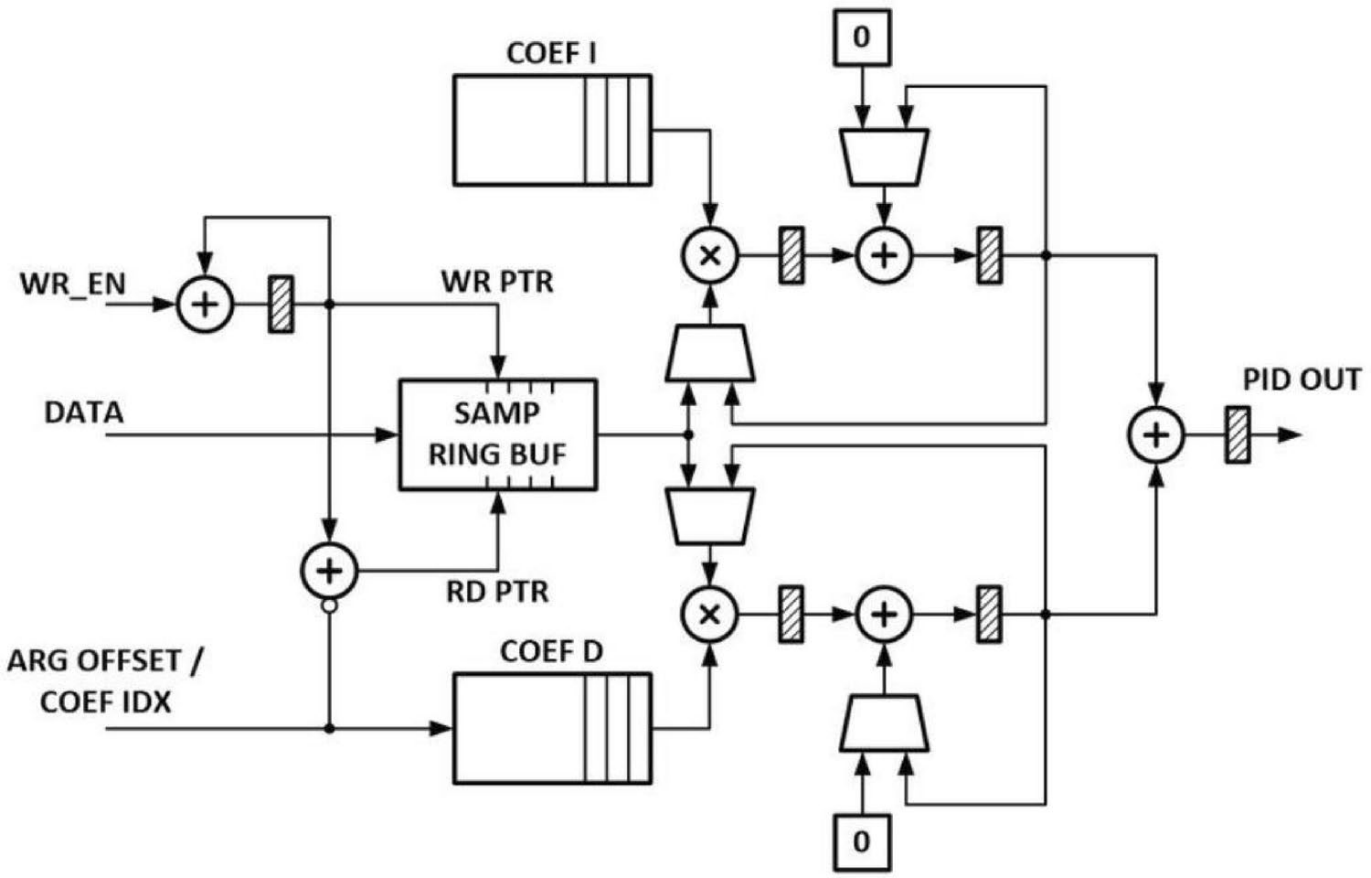
FPGA design for ${\textrm{PI}}^\lambda {\textrm{D}}^\mu$ controller.

Figure 2 presents a simplified version of the created FPGA design. To speed up the calculations, the calculation unit uses the elements specific to the FPGA we have used.

In the proposed design, the coefficients and queue length samples are stored in the BRAM memory, allowing the easy scalability of the buffer sizes. The content of the memory is initialized during the configuration; hence, it is possible to transfer the testing samples to the devices during the configuration phase. Test queue length samples are stored in the ring buffer. One of its gates is used to write the samples to the buffer while the other reads the samples for the memory block. The read of the samples is done based on the indexed access. From the base address, the index is subtracted to determine the exact element from memory to access at the given moment. Consequently, each write of the coefficient causes a shift of the memory by one element.

The arithmetic unit is built of accumulators. At the first step of the calculation process, the accumulators are used to calculate the values of integral and derivative terms of the controller. Then, the values are multiplied by the corresponding amplification factors, accumulators are initialized, and the proportional term of the controller is calculated based on the most recent queue length sample. At last, the calculated values are added up to calculate the overall controller response.

The arithmetic path was designed for the complete utilization of available multiplication units. Calculations are done on 35-bit signed integers. The accumulator was extended to 48 bits to prevent overflows. We have used specialized DSP48E1 modules in the implementation.

The time complexity of the calculation process is *n + 4* tacts of the clock signal, where *n* is a number of coefficients. The maximum frequency of the clock is 100 MHz. One crucial observation is the possibility of quick pre-calculations based on the old samples before the new sample’s arrival. In such a case, six tacts of the clock signal are enough to calculate the controller’s response.

## Methods

We divided experiments into two stages to examine calculation error influence on controller efficiency. The main goal of the first stage, conducted in the simulation environment, was to compare the behavior of the benchmark implementation, which utilizes floating point operations implemented in Python, to the implementation based on the estimated approach. The created AQM algorithm was validated in the testbed environment in the second research stage.

The first stage of research was conducted in two steps. In the first step, the packet drop probability was compared for the benchmark and estimated-based approach solution using the dataset of queue length samples. The dataset contained 10000 samples of values in the range from 1 to 100, generated using a random numbers generator of uniform distribution. In the second step, we compared the behavior of both solutions with the SimPy library. The simulator used in the experiments was the system composed of one packets producer who puts generated packets in the queue and one consumer who consumes packets from it. The queue is supervised by an AQM algorithm. On the packet’s enqueue event, the controller calculates ${\textrm{PI}}^\lambda {\textrm{D}}^\mu$ value, which is used as dropping probability and gathers the statistics about the amount of processed and dropped packets to calculate the percentage of early dropped packets.

**Figure 3:**
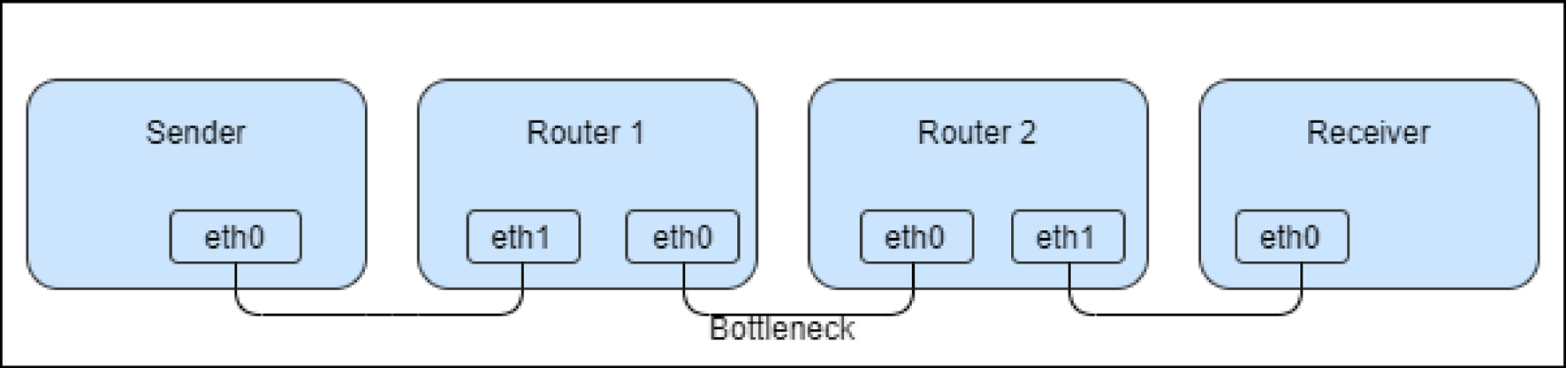
Network topology used in testbed.

The second research stage was conducted using a testbed created with physical devices. Testbed was created using the guidelines provided in RFC7928^17^. The experiment testbed consists of two Linux-based machines and two routers Mikrotik Routerboard RB951UI-2HND as shown in Fig. 3.

With many AQM algorithms comes the problem of optimal adjustment of their parameters for a given network configuration. Furthermore, with traffic conditions in the given network varying over time, the correct parameters setup may also change. The correct parameters choice is affected by properties of network traffic, like it’s intensity or self-similarity^32^. The influence of parameters on the dropping probability function was presented in earlier works^33^. Therefore, one of the research goals was to find such parameters setup for which the average queue length and the number of dropped packets are close to minimal. Such a setup should decrease the delay experienced by packets without significant loss in available bandwidth.

${\textrm{PI}}^\lambda {\textrm{D}}^\mu$ controller parameters for each experiment for all of the conducted experiments are presented in table 1. Due to the calculational capabilities of the router, as well as due to calculation error introduced with the alternative calculations method, we use the length of error history of 5 as optimal for our needs.

**Table 1:** Experiments parameters.

Parameter	Examined parameter				
	$K_p$	$K_i$	$K_d$	$\lambda$	$\mu$
Limit	100	100	100	100	100
Setpoint	50	50	50	50	50
Error history	5	5	5	5	5
$K_p$	–	0	0	0	0
$K_i$	0	–	0	0.001	0
$K_d$	0	0	–	0	0.001
$\lambda$	− 0.1	− 0.1	− 0.1	–	− 0.1
$\mu$	0.8	0.8	0.8	0.8	–

Modified system OpenWRT with ${\textrm{PI}}^\lambda {\textrm{D}}^\mu$ algorithm was installed and set up on the device Router 1. The other router in the network used the original producer’s system Mikrotik RouterOS . As shown in Fig. 3, the bottleneck in the network was the connection between routers. We use iPerf3 with parallel TCP connections to generate traffic from sender to receiver. Additionally, every 5 ms was generated TCP packet, and its RTT time was measured using hping3. To achieve stable network traffic, each experiment lasted for 30 s. To ensure repeatable results, each experiment was performed ten times. Experiments were conducted in three different traffic conditions in a testbed environment. For light traffic, we used 3 parallel TCP connections, for moderate 10 connections and for heavy—25 connections.

## Results

In this section, we present the results of conducted experiments. In the subsection “Numerical analysis and simulation results”, we present the results of numerical analysis and simulation results of the proposed integer-based ${\textrm{PI}}^\lambda {\textrm{D}}^\mu$ controller compared to benchmark implementation using floating-point operations. In subsection “Testbed results”, we present the results of the AQM examination in the created testbed environment. Controller parameters used in experiments were presented in the Table 1, and a detailed description of the methodology of the experiments is provided in Section “Methods”.

### Numerical analysis and simulation results

A numerical analysis of the created controller is presented in “Numerical analysis and simulation results”, during which the probability of dropping a packet was examined. The same research was also conducted with a simulation environment, the results of which are presented in “Numerical analysis and simulation results”.

#### Dropping probability comparison

**Figure 4:**
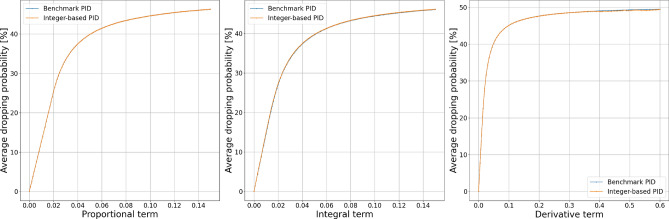
Comparison of dropping probability.

**Figure 5:**
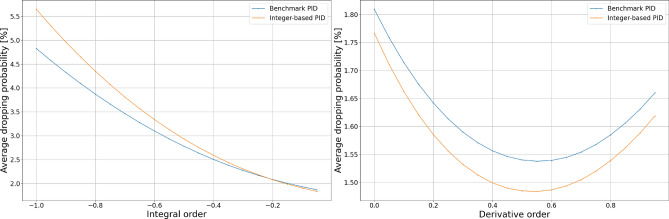
Comparison of dropping probability.

The results of fixed dataset simulation experiments presented in Figs. 4 and 5 show that the alternative calculation method does not impact the controller dropping probability for proportional, integral, or derivative terms. However, we may notice differences in behavior for both essential and derivative orders.

#### Packets dropped comparison

**Figure 6:**
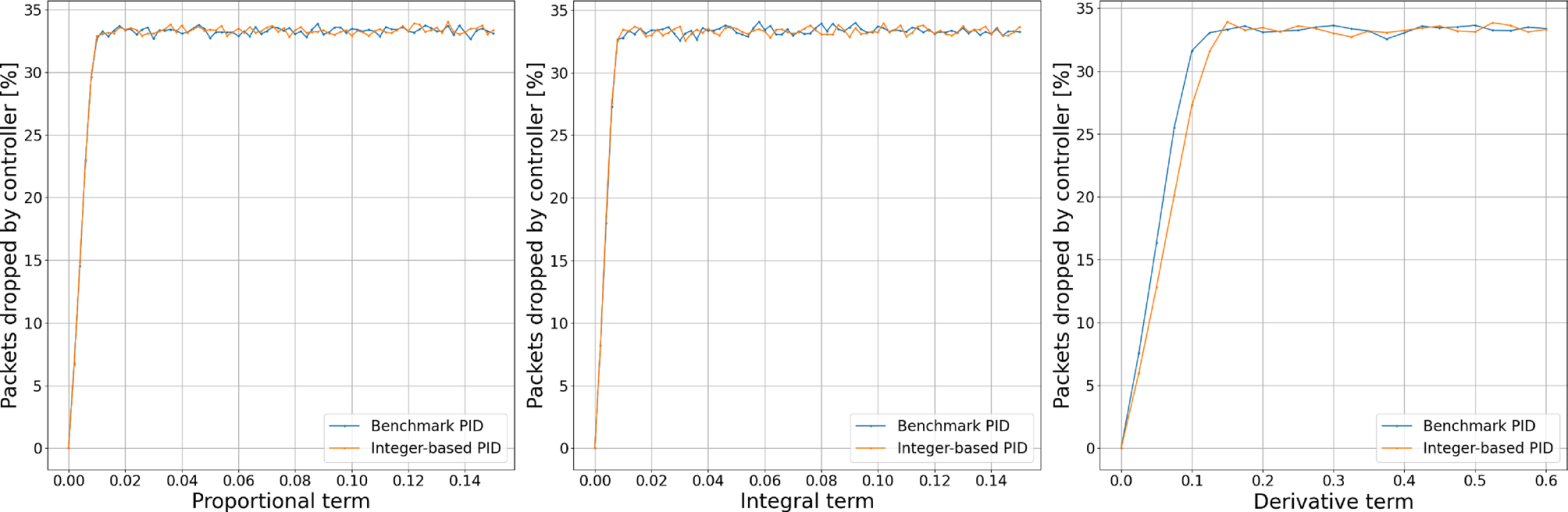
Packets dropped by controller.

**Figure 7:**
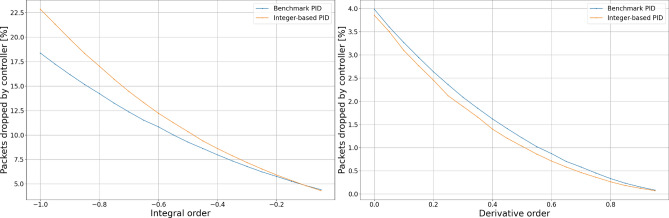
Packets dropped by controller.

Having the comparison of dropping probability, we conducted experiments to determine the impact of calculation error introduced with an alternative calculation method on the number of packets dropped by the controller. To achieve growing queue sizes over time, the packets producer produces 1.5 packets per time unit while the consumer consumes 1.0 packets per time unit. Results of the experiments in terms of packets dropped by the controller are shown in Figs. 6 and 7.

We can notice that experiment results obtained with the simulator are consistent with those obtained with the fixed dataset. We can also observe that proportional and integral terms are the biggest contributors to the number of dropped packets by the controller. The number of packets dropped by the controller grows slower with increasing derivative term, making this parameter especially useful for the precise setup of the controller. Using fractional order ${\textrm{PI}}^\lambda {\textrm{D}}^\mu$ controller, we can achieve even more subtle controller’s behavior setup. Even though the proposed calculation method introduces a difference in the amount of dropped packets, this controller feature can still achieve better performance in given network conditions; however, it requires a slightly different setup.

### Testbed results

The key to AQM effectiveness is careful parameters setup for the given working environment. There is a need for finding close to optimal balance between decreasing the delay (via dropping packets) and limiting the bandwidth (due to required retransmissions). To perform such a setup in our testbed, we have conducted several experiments on the controller’s parameters’ influence on dropped packets, presented in “Testbed results”. Then, we have examined how the packets dropping affects packets round-trip time and throughput in the network, presented in “Testbed results”. Finally, using the results from previous steps, we have compared the proposed AQM to the FIFO queue, presented in “Testbed results”.

#### Packets dropped

To test the influence of each controller’s parameters on the number of packets being dropped during the transmission, we distinguish two measures: packets dropped exclusively by the AQM algorithm and the total number of packets dropped—by either queue overflow or the AQM algorithm.

**Figure 8:**
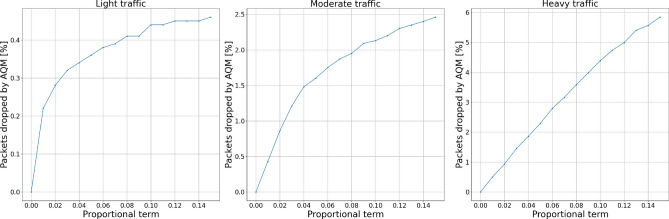
Packets dropped by the AQM—proportional term.

**Figure 9:**
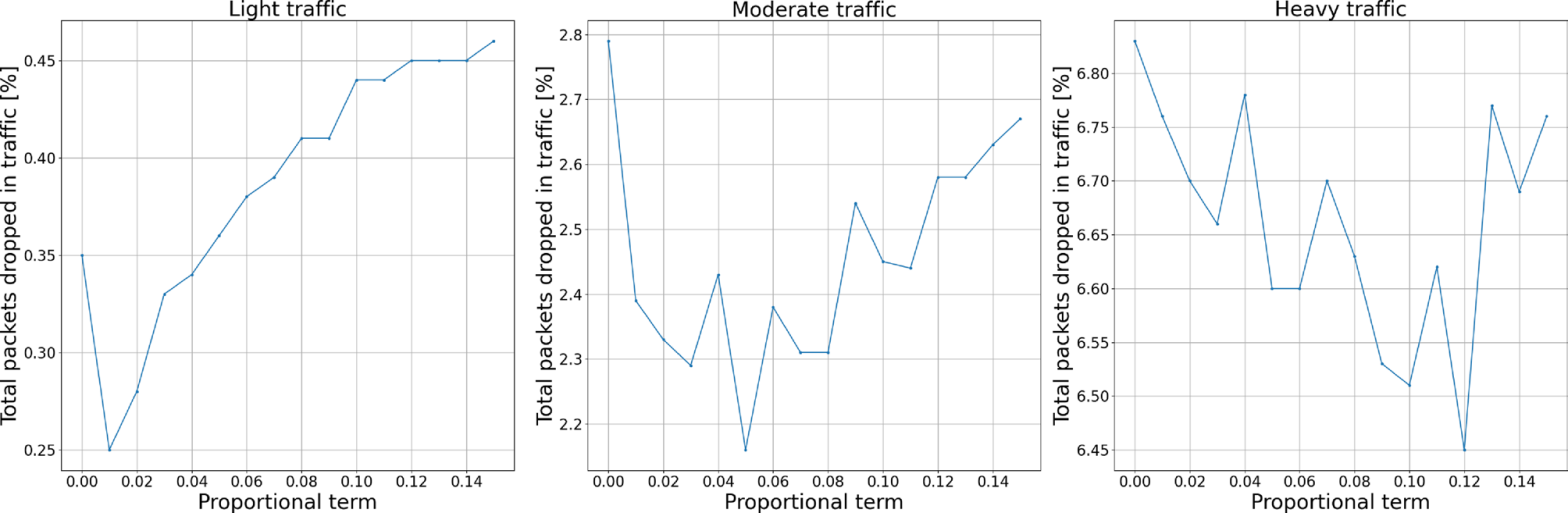
Total dropped packets—proportional term.

Figures 8 and 9 show the experiment results for proportional term changes. We can see a growing number of packets dropped by the AQM with the growing value of the parameter. It is worth noticing that the number of packets dropped by the AQM increases with the traffic intensity.

**Figure 10:**
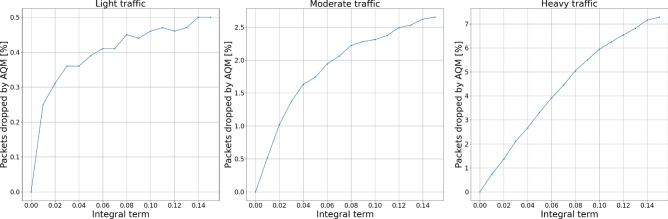
Packets dropped by the AQM—integral term.

**Figure 11:**
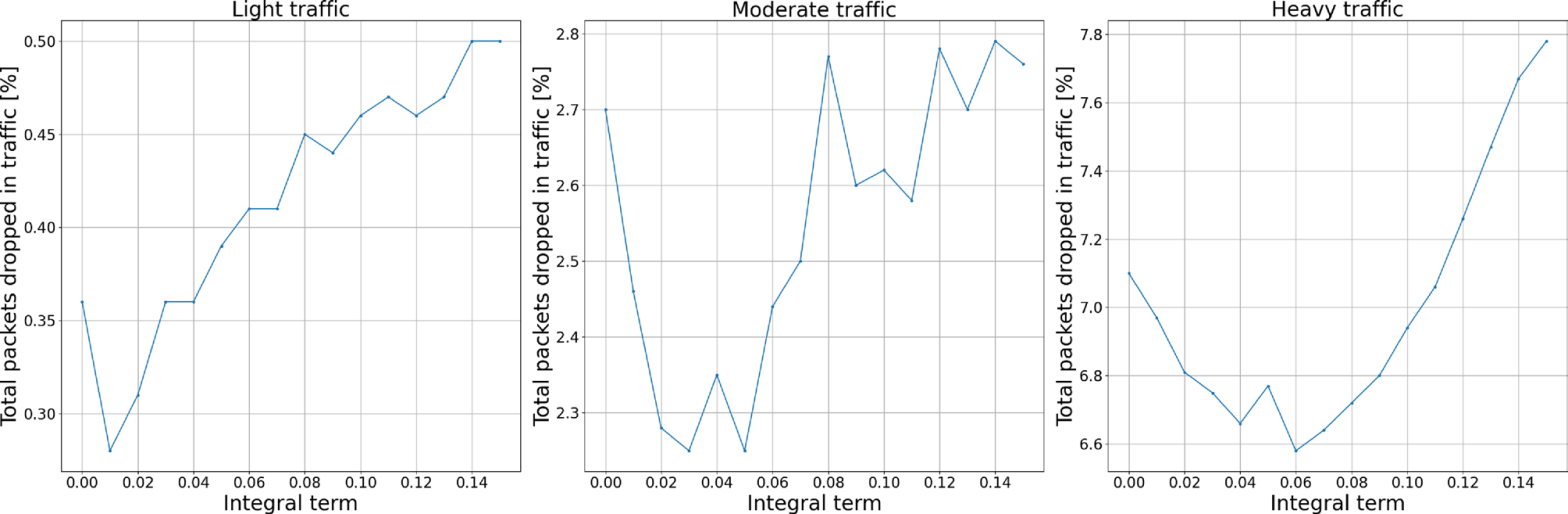
Total dropped packets—integral term.

Figures 10 and 11 show the experiment results for integral term changes. We can observe that this parameter behaves similarly to the proportional term. However, with flexibility given by the change of integral order, its behavior may be tuned more flexibly, which will be presented further.

**Figure 12:**
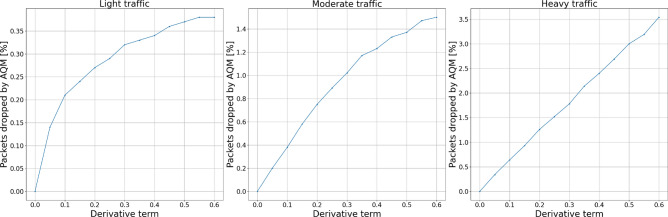
Packets dropped by the AQM—derivative term.

**Figure 13:**
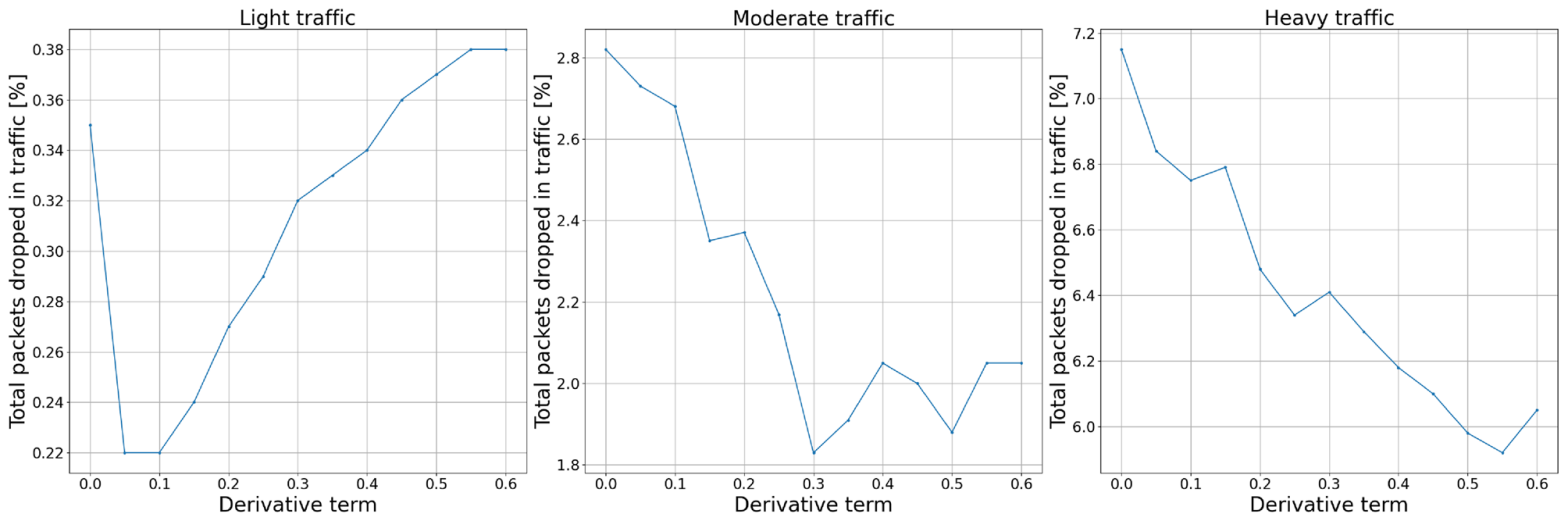
Total dropped packets—derivative term.

Figures 12 and 13 show the experiment results for derivative term changes. We can see that the derivative part of the controllers drops fewer packets than the previously described proportional and integral terms. This controller feature, together with the even more subtle influence of changing derivative order, allows for more flexible tuning of the controllers to the specific network requirements.

**Figure 14:**
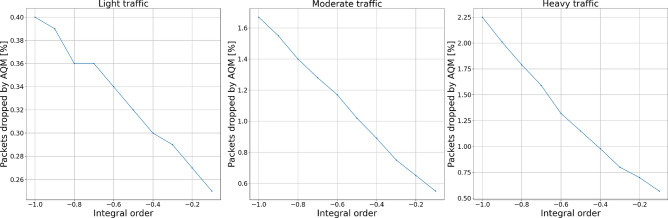
Packets dropped by the AQM—integral order.

**Figure 15:**
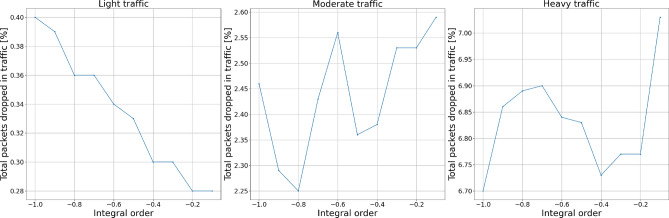
Total dropped packets—integral order.

**Figure 16:**
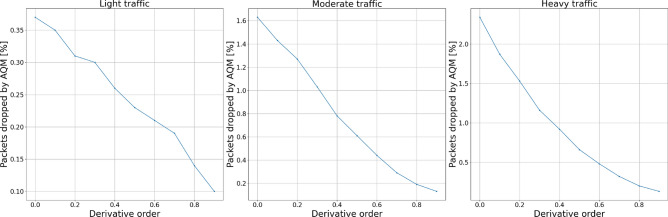
Packets dropped by the AQM—derivative order.

**Figure 17:**
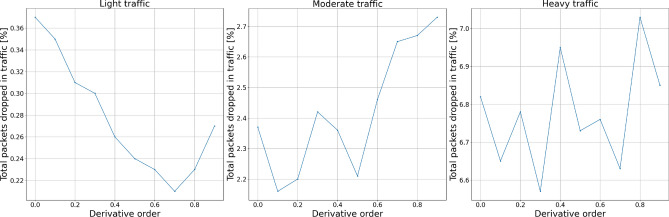
Total dropped packets—derivative order.

Figures 14, 15, 16 and 17 show the influence of the integral and derivatives orders on the number of dropped packets. We can see that changing orders for integral and derivative terms of the controller enables the possibility for a subtle tuning of the AQM behavior for the given amplification value. For certain scenarios, changes in the amplification values may lead to either too high dropping probability or too low. In such cases, changes in the order of one of the fractional order terms allow correct controller tuning.

Looking at the results, we may notice a local minima for the total dropped packet number. This means that even though the AQM mechanisms drop some packets without the urgent need to do so, the overall stability of the network increases. Such local minima are the key to proper tuning of AQM for the given network under certain traffic conditions.

#### Delay in network and throughput

**Figure 18:**
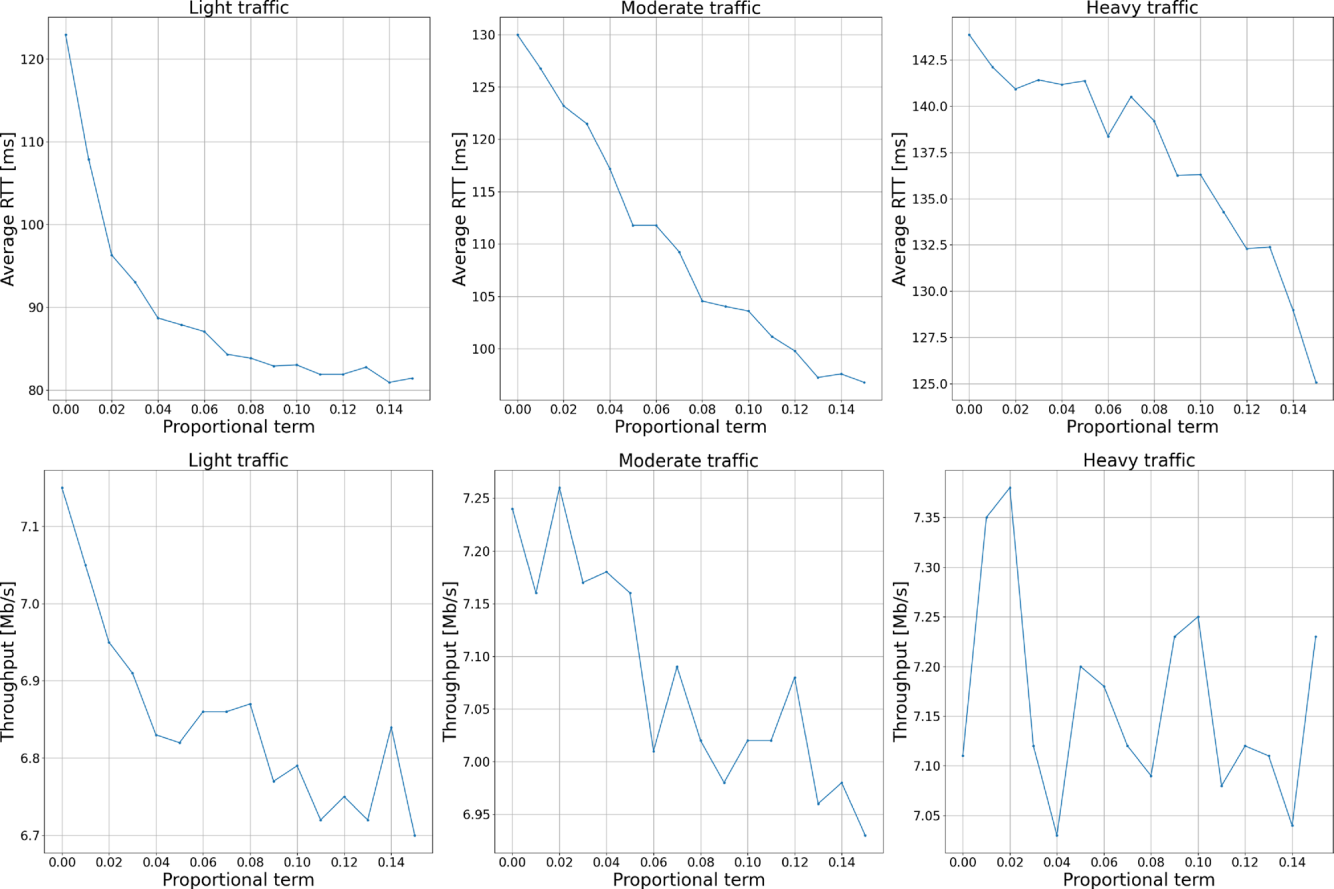
Packet’s round-trip time and throughput in the network.

Knowing the influence of the parameter’s packets dropped, we have examined the influence of dropped packets on the delay and throughput of the network. Figure 18 presents the round-trip time and throughput as a function of the proportional term. The presented results show that increasing the number of dropped packets leads to the delay decrease on the one hand but to throughput degradation on the other. However, we can see a certain improvement in the delays, which can be achieved without significant loss in bandwidth, especially for more congested traffic conditions.

#### FIFO and ${\textrm{PI}}^\lambda {\textrm{D}}^\mu$ comparison

In the last stage of experiments, we compared the delay experienced by packets and network bandwidth between the proposed AQM mechanism and the traditional FIFO queue. We have used the data collected in the earlier stages to correctly set ${\textrm{PI}}^\lambda {\textrm{D}}^\mu$ controller parameters for each tested scenario. Experiment parameters are presented in table 2.

**Table 2:** ${\textrm{PI}}^\lambda {\textrm{D}}^\mu$ controller parameters.

Parameter	Light traffic	Moderate traffic	Heavy traffic
Limit	100	100	100
Setpoint	50	50	50
Error history	5	5	5
$K_p$	0	0.035	0.08
$K_i$	0	0.02	0.05
$K_d$	0.006	0.3	0.4
$\lambda$	− 0.1	− 0.2	− 0.2
$\mu$	1.0	0.7	0.7

**Figure 19:**
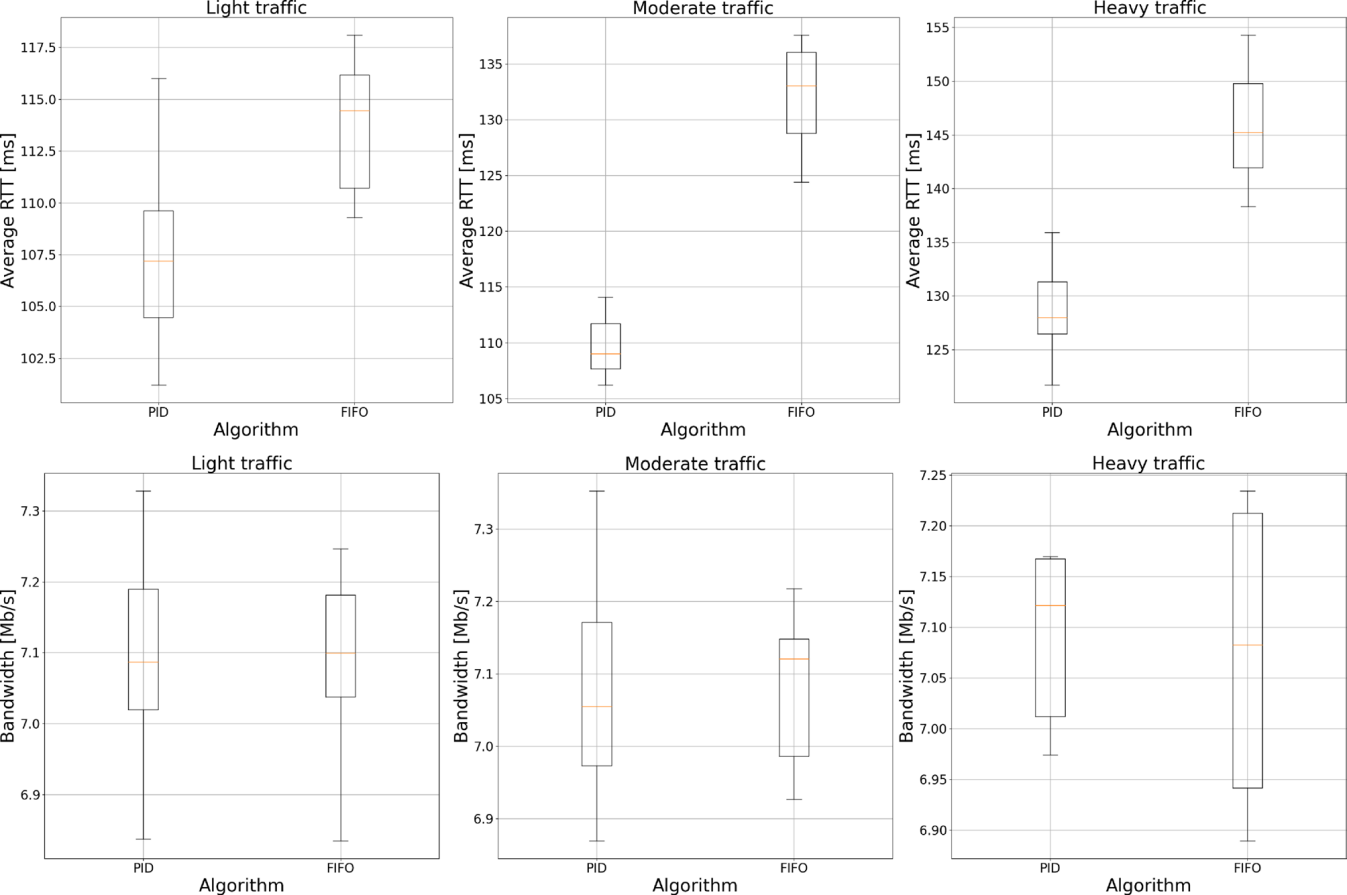
${\textrm{PI}}^\lambda {\textrm{D}}^\mu$ and FIFO comparison.

Results of the experiments are shown in Fig. 19. Looking at the results, we can notice that with the created algorithm, we can improve the delay experienced by packets in all tested cases compared to the standard FIFO queue without a noticeable negative impact on the bandwidth. These results show that we have been able to create the working solution and propose the process of its setup for the given network configuration.

## Conclusions

The research aimed to recognize the implementation limitations related to using fractional order ${\textrm{PI}}^\lambda {\textrm{D}}^\mu$ controller as AQM mechanism. Due to a challenge related to floating point operations, we have proposed an alternative approach to the calculation process and tested this approach in simulation and real-life routers. Besides that, we created an FPGA design for speeding up the calculation process. The implementation limitations of usage fractional order ${\textrm{PI}}^\lambda {\textrm{D}}^\mu$ as AQM mechanism have been recognized. The alternative approach of calculating the controller’s response introduces the calculation error in the process but does not disqualify this approach from real-life applications. The proposed solution still serves its purpose of controlling the delay in the TCP-based network, allowing it to decrease the delay experienced by the traffic in the network without crippling the available bandwidth. Further improvement in the controller’s behavior can be achieved by using FPGA, which can further speed up the calculation process. By changing the size of the calculation units in FPGA, we can also eliminate the problem of a calculation error, enabling the possibility of enlarging the errors history to gain a more precise controller’s reaction to the conditions in the network. We have validated the solution in simulation and testbed environments. Using a simulation environment, the integer-based solution was compared to the benchmark solution, which utilizes floating-point operations to conduct calculations. In the testbed environment, ${\textrm{PI}}^\lambda {\textrm{D}}^\mu$ was used as an AQM algorithm, and its performance in decreasing delay experienced by packets in the network was compared to standard FIFO queue. The setup of the controller’s parameters for the given network is not a trivial problem, so we have shown a way of finding the optimal setup of the controller’s parameters to improve the quality of services in the network. Enhancing the controller with the auto-tuning feature is another way of further improving it’s performance in the network.

### Supplementary Information

Supplementary Information.

## Data Availability

We include data used to generate figures presented in the article in the Supplementary Materials folder. The same data will be published on our GitHub repository upon acceptance.
